# Non-invasive Imaging and Modeling of Liver Regeneration After Partial Hepatectomy

**DOI:** 10.3389/fphys.2019.00904

**Published:** 2019-07-17

**Authors:** Sara Zafarnia, Anna Mrugalla, Anne Rix, Dennis Doleschel, Felix Gremse, Stephanie D. Wolf, Johannes F. Buyel, Ute Albrecht, Johannes G. Bode, Fabian Kiessling, Wiltrud Lederle

**Affiliations:** ^1^Institute for Experimental Molecular Imaging, Medical Faculty, RWTH Aachen University, Aachen, Germany; ^2^Department of Gastroenterology, Hepatology and Infectious Diseases, Medical Faculty, Heinrich-Heine-University Düsseldorf, Düsseldorf, Germany; ^3^Fraunhofer Institute for Molecular Biology and Applied Ecology IME, Aachen, Germany; ^4^Institute for Molecular Biotechnology, RWTH Aachen University, Aachen, Germany

**Keywords:** non-invasive imaging, modeling, liver regeneration, partial hepatectomy, macrophages, angiogenesis, FMT-μCT

## Abstract

The liver has a unique regenerative capability upon injury or partial resection. The regeneration process comprises a complex interplay between parenchymal and non-parenchymal cells and is tightly regulated at different scales. Thus, we investigated liver regeneration using multi-scale methods by combining non-invasive imaging with immunohistochemical analyses. In this context, non-invasive imaging can provide quantitative data of processes involved in liver regeneration at organ and body scale. We quantitatively measured liver volume recovery after 70% partial hepatectomy (PHx) by micro computed tomography (μCT) and investigated changes in the density of CD68^+^ macrophages by fluorescence-mediated tomography (FMT) combined with μCT using a newly developed near-infrared fluorescent probe. In addition, angiogenesis and tissue-resident macrophages were analyzed by immunohistochemistry. Based on the results, a model describing liver regeneration and the interactions between different cell types was established. *In vivo* analysis of liver volume regeneration over 21 days after PHx by μCT imaging demonstrated that the liver volume rapidly increased after PHx reaching a maximum at day 14 and normalizing until day 21. An increase in CD68^+^ macrophage density in the liver was detected from day 4 to day 8 by combined FMT-μCT imaging, followed by a decline towards control levels between day 14 and day 21. Immunohistochemistry revealed the highest angiogenic activity at day 4 after PHx that continuously declined thereafter, whereas the density of tissue-resident CD169^+^ macrophages was not altered. The simulated time courses for volume recovery, angiogenesis and macrophage density reflect the experimental data describing liver regeneration after PHx at organ and tissue scale. In this context, our study highlights the importance of non-invasive imaging for acquiring quantitative organ scale data that enable modeling of liver regeneration.

## Introduction

The liver is known for its high regenerative potential being able to restore up to 70% of its mass after injury or partial resection (Minuk, 2003). The regeneration process constitutes a complex interplay of various cell types and signaling pathways (Taub, 2004). Depending on the circumstances, two different modes of regeneration are known to be activated. In case of an impaired hepatocyte proliferation, as for instance following severe or chronic liver injury, liver stem cells (also known as oval cells in rodents) become activated as a mechanism of the liver to regenerate and recover its function (Itoh and Miyajima, 2014). In contrast, after partial resection or moderate liver damage, complete liver regeneration is achieved by proliferation of the remaining parenchymal and non-parenchymal cells. Hepatocytes are the first cells to grow and proliferate after partial resection followed by Kupffer cells, biliary epithelial cells, and stellate cells. The process is accompanied by the induction of angiogenesis that is also crucially involved in liver regeneration (Drixler et al., 2002; Uda et al., 2013). Besides other cell types, macrophages have been shown to play a stimulatory role in liver regeneration by producing molecular factors that are pivotal in the regeneration process (Abshagen et al., 2007; Li and Hua, 2017). Depletion of resident macrophages (Kupffer cells) as well as an impaired macrophage recruitment from the periphery and bone marrow results in a delayed regeneration demonstrating the stimulatory function of both resident and infiltrating macrophages (Takeishi et al., 1999; Abshagen et al., 2007; Melgar-Lesmes and Edelman, 2015; Nishiyama et al., 2015). The most frequently used model to study liver regeneration is the model of partial hepatectomy (PHx) described first by Higgins and Anderson (1931) in rats. Mitchell and Willenbring recently developed a modified protocol of a standardized surgical technique for PHx in mice (Mitchell and Willenbring, 2008). In both models, approximately two-thirds of the liver are surgically removed. The regeneration process starts immediately leading to full recovery of liver mass within 7–10 days (Taub, 2004; Nishiyama et al., 2015). The advantage of a surgical model in comparison with toxic injury models is the fact that the regeneration process after PHx is not associated with massive necrosis and necrosis-induced acute inflammation so that all changes observed after PHx can be ascribed to the physiological regeneration process (Michalopoulos, 2010). In a clinical context, this physiological regeneration process becomes important in patients who underwent partial liver resection, in donors and recipients following living-donor liver transplantation and in patients with acute liver failure.

Liver regeneration is a complex process involving the interactions of different cell types on various levels. Thus, we followed an approach using multi-scale methods (Castiglione et al., 2014) including non-invasive imaging and histological analyses in order to investigate liver regeneration after pHx. Non-invasive imaging is a useful tool since it enables a longitudinal and quantitative assessment of morphological, functional, and molecular parameters at the organ and whole body level. Liver volume recovery was measured *via* micro computed tomography (μCT), and the density of CD68^+^ macrophages was determined by combined fluorescence-mediated tomography and μCT (FMT-μCT) using a newly developed near-infrared fluorescent (NIRF) probe. The *in vivo* results were validated by immunohistochemical analyses of CD68^+^ and F4/80^+^ macrophages. At the tissue level, the contribution of tissue-resident macrophages and angiogenesis was investigated by additional immunohistochemical analyses. Based on the experimental data, a simple model describing liver regeneration and the interrelation between volume recovery, macrophages, and angiogenesis was generated.

## Materials and Methods

### Generation and Purification of the Near-Infrared Fluorescent CD68 Probe

The NIRF probe targeting CD68^+^ macrophages was generated by coupling an amine-reactive NIR fluorochrome (NHS ester), VivoTag 680 (excitation peak 665 ± 5 nm, emission peak 688 ± 5 nm) (Perkin-Elmer), to a rat anti-mouse CD68 antibody (AbDSerotec) according to manufacturer’s instructions. In brief, VivoTag 680 was dissolved in dry dimethyl sulfoxide (Sigma-Aldrich) at a concentration of 10 mg/ml. Prior to the labeling, the buffer of the antibody was exchanged by dialysis into conjugation buffer (50 mM carbonate/bicarbonate buffer, pH 8.5) using Slide-A-Lyzer dialysis cassettes (AbD Serotec) according to the protocol provided by the manufacturer. After buffer exchange, 30 μl of VivoTag 680 was added to the rat anti-mouse CD68 antibody. Following 1 h of incubation at room temperature protected from the light, the NIRF CD68 probe (approximately 151 kDa) was separated from free fluorescent dye (approximately 1 kDa) and antibody oligomers (larger than 300 kDa) by fast protein liquid chromatography using a Superdex 200 resin in a pre-packed 10/300 GL column (GE Healthcare). Probe concentration was determined using a BCA Protein Assay Kit (Uptima) according to the protocol provided by the manufacturer.

### *In vitro* Binding of the Near-Infrared Fluorescent CD68 Probe

Binding specificity of the NIRF CD68 probe was tested *in vitro* by incubating the macrophage cell line J774A.1 (CLS Cell Lines Service) with the NIRF CD68 probe (10 nM, 2 or 4 h, 37°C). For competitive binding analyses, J774A.1 cells were incubated with 10 nM of the NIRF CD68 probe and a 10-fold molar excess of unlabeled CD68 antibody (100 nM, 2 or 4 h, 37°C). Fluorescent microphotographs were acquired with the Axio Imager M2 (Zeiss) and a high-resolution camera (AxioCamMRm Rev.3; Zeiss) using a Cy5.5 filter and a fixed exposure time. The signal intensities were determined using the software ImageJ 1.47v (W. Rasband, National Institutes of Health). For quantification, the mean fluorescent signal intensity at 695 nm was determined by analyzing five microscopic images per well (*n* = 3 wells per culture condition).

### Animal Studies

All animal experiments were performed according to German legal requirements and animal protection laws and were approved by the Authority for Environment Conservation and Consumer Protection of the State of North Rhine-Westphalia (LANUV).

### Biodistribution and *in vivo* Specificity of the Near-Infrared Fluorescent CD68 Probe

Biodistribution and *in vivo* specificity of the NIRF CD68 probe were analyzed in male C57BL/6 J mice (Charles River) (*n* = 5 per group). The mice were fed a chlorophyll-free diet (ssniff Spezialdiäten GmbH) 7 days before imaging, and the scanning area was depilated prior to the scans. For macrophage imaging, 2.6 μg of the NIRF CD68 probe dissolved in 0.9% w/v NaCl was injected intravenously (i.v.). To analyze the biodistribution, animals were scanned longitudinally immediately before and 1, 3, 6, 12, 24, and 48 h after probe injection by FMT-μCT. *In vivo* specificity of the probe was examined by competitive binding analysis injecting a 5-fold mass excess of unlabeled CD68 antibody (13 μg) intraperitoneally (i.p.) 1 h before i.v. injection of the NIRF CD68 probe (2.6 μg). Directly after the last FMT-μCT measurement, the mice were sacrificed, and the liver was resected and cryoconserved in Tissue-Tek (Sakura) for immunohistochemical analyses.

### *In vivo* Determination of Macrophage Density and Volume Recovery During Liver Regeneration After Partial Hepatectomy

Liver regeneration was analyzed in male C57BL/6 J mice (Charles River) after PHx and sham surgery. One hour before surgery, mice were treated with carprofen [subcutan (s.c.) 5 mg/kg] (Pfizer Animal Health SA). PHx and sham surgeries were performed under sterile conditions as previously described by Mitchell and Willenbring (2008). In brief, mice were anesthetized with isoflurane (2% v/v isoflurane in oxygen-enriched air) and positioned on a temperature-controlled pad to regulate body temperature. For PHx, after midline laparotomy, the left lateral lobe was ligated as close to the base of the lobe as possible. The secondary knot was placed above the gall bladder of the median lobe but not closer than 2 mm from the suprahepatic vena cava. The ligated liver lobes were surgically resected. At the end of the surgery, the abdomen was rinsed with saline solution, and the abdominal wall and the skin were sutured separately. For sham surgery, a midline laparotomy was performed with gentle palpation and manipulation of the liver without resection of the liver lobes. Directly after surgery, mice received a s.c. injection of 10 mg/kg enrofloxacin (Bayer). Afterwards, analgesia was continued by s.c. injection of 5 mg/kg carprofen (Pfizer Animal Health SA) once per day for 3 days.

CD68^+^ macrophages and volume recovery were monitored by FMT-μCT and contrast-enhanced μCT at different time points (4, 8, 14, and 21 days) after PHx and sham surgery. As an additional control, untreated mice were measured. Twelve hours before each FMT-μCT measurement, mice were injected i.v. with 5.7 μg of the NIRF CD68 probe diluted in 0.9% w/v NaCl. In addition, 45 min before the FMT-μCT scans, the mice received an i.v. injection (150 μl) of the contrast agent Imeron 400 (Bracco Imaging) to enable a better segmentation of the liver. Directly after each FMT-μCT measurement, mice were sacrificed, and the liver was resected and cryoconserved in Tissue-Tek (Sakura) for immunohistochemical analyses. The group size was as follows: for assessment of macrophage density: untreated mice: *n* = 10; mice after PHx: *n* = 5 for day 4, *n* = 4 for day 8, *n* = 4 for day 14, *n* = 4 for day 21; sham-operated mice: *n* = 3 for day 4, *n* = 3 for day 8, *n* = 3 for day 14, *n* = 6 for day 21. For volume recovery analysis: *n* = 5 for each time point.

### Imaging Protocols

Three-dimensional (3D) FMT-μCT scans were conducted as described by Kunjachan et al. (2013). For the measurements, mice were anesthetized and positioned in a μCT- and FMT-compatible mouse bed. For anatomical information, mice were scanned in a dual-energy μCT system (TomoScope 30s Duo, CT Imaging GmbH). For biodistribution and *in vivo* competitive binding, analyses scans were performed using the scan protocol SQD-6565-360-29, which acquires 720 projections with 516 × 506 pixels requiring a scanning time of 29 s per subscan. For the assessment of macrophage density and liver volume, the HQD-6565-360-90 protocol was applied, which acquires 720 projections with 1,032 × 1,012 pixels requiring a scanning time of 90 s per subscan. Directly after acquiring the μCT scans, the mouse bed was transferred to the FMT system (FMT 2500LX, PerkinElmer), and FMT scans were performed at 680 nm using 120 excitation positions (3 mm distance). Data fusion and reconstruction of the fluorescence distribution were performed as described (Gremse et al., 2014). Based on the μCT data, organs were manually segmented, and the liver volume and probe concentration in the segmented organs were determined using the Imalytics Preclinical software (ExMI/Gremse-IT) (Gremse et al., 2016). To assess the variability of the organ segmentations, several organs (liver, lung, heart, kidney, and bladder) were segmented in five representative scans by two different persons. The organ volumes of the two analyses correlated strongly (*R*^2^ = 0.996, *p* < 0.05) between the users. DICE scores, which describe similarity between segmentations, were also high (0.92 ± 0.045), showing good reproducibility of the manual organ segmentation. To further confirm the accuracy of manual organ segmentation, we performed an additional correlation analysis between segmented organ volumes and weights of excised organs. Organ weights (heart, liver, kidneys, spleen, and tumors) and contrast-enhanced μCT scans (*n* = 6) were available from a previous study (Rosenhain et al., 2018). The analysis resulted in a strong correlation between segmented organ volumes from contrast-enhanced μCT scans and the organ weights (*R*^2^ = 0.976). The slope of the regression line was 0.84, i.e., below 1, which can be explained by the loss of blood during the organ excision and harvesting procedure (thus lower values for the organ weights as compared to the segmented volumes).

### Antibodies

The following primary antibodies were used to stain macrophages/Kupffer cells: rat anti-mouse CD68 antibody (AbDSerotec), rat anti-mouse F4/80 antibody (AbDSerotec), and rat anti-mouse CD169 antibody (AbDSerotec). To stain endothelial cells, rat anti-mouse CD31 antibody (BD Biosciences) was applied. Goat anti-mouse VEGFR2 antibody (R and D Systems) was used to determine the VEGFR2 density. Secondary IgG antibodies (donkey anti-rat Alexa Fluor 488, donkey anti-rat Cy-3 and donkey anti-goat Cy-3) were obtained from Dianova. Cell nuclei were counterstained with 4′,6-diamidino-2-phenylindole (DAPI; Merck KGaA).

### Indirect Immunohistochemistry

For immunohistochemical analysis, frozen organs were cut into 8-μm slices. Fixation and staining of the cryosections were performed as previously described (Lederle et al., 2010). Per section, five to seven fluorescent microphotographs were acquired with the Axio Imager M2 (Zeiss) and a high-resolution camera (AxioCamMRm Rev.3; Zeiss). The number of CD68^+^, F4/80^+^, CD169^+^ and DAPI^+^ cells per microphotograph was counted manually using the ImageJ 1.47v software (W. Rasband, National Institutes of Health), and the percentage of CD68^+^, F4/80^+^ and CD169^+^ cells per DAPI^+^ cells was calculated, respectively. Quantitative analysis of microvessel density and angiogenic activity was done using the AxioVisionRel 4.8 software (Zeiss). The microvessel density was determined by quantifying the CD31^+^ area fraction, and the angiogenic activity was assessed by determining the proportion of the VEGFR2^+^ area fraction to the CD31^+^ area fraction. The group size was as follows: untreated mice: *n* = 10 (CD169: *n* = 5); mice after PHx: *n* = 5 for day 4, *n* = 4 for day 8, *n* = 4 for day 14, *n* = 4 for day 21; sham-operated mice: *n* = 4 for day 4, *n* = 6 (CD169: *n* = 3) for day 8, *n* = 3 for day 14, *n* = 7 for day 21.

### Numeric Modeling

Stimulation and growth of liver cell compartments (hepatocytes, Kupffer cells, macrophages, and endothelial cells) were described by differential equations in a simplified model. Based on data from literature, relative proportions of these cell types in healthy livers were assumed to be 0.8, 0.06, 0.06, and 0.08, respectively (Vekemans and Braet, 2005). To simulate PHx, these values were multiplied by 0.3 at the initial state of simulation to account for the reduced total cell amount. The first derivative of each cell compartment fraction was assumed to depend linearly on the amount, excess, or lack of other cell compartments, with six coefficients describing the strength of the corresponding effect. The parameters *k*_HE_ and *k*_ME_ describe the stimulation and support of vessel growth by a hepatocyte lack and macrophage excess, respectively. *k*_HM_ describes attraction of macrophages by a lack of hepatocytes. *k*_HK_ describes stimulation of Kupffer cell growth by a lack of hepatocytes. *k*_EH_ describes the connection of blood vessel and hepatocyte growth, because the latter is limited by nutrition supply and structural alignment requirements. A parameter *k*_homeostasis_ describes other effects not covered by our simplified model, and it drives the four cell compartments slowly toward the homeostatic situation. Therefore, the derivatives of the four compartments are the weighted sums of the affecting coefficients and expressions for relative or absolute lack as described in the following:

$$dH\;/\;dt\;=\;k_{\mathrm{homeostasis}}\left(1\;-\;H\;/\;0.8\right)\;+\;k_{\mathrm{HE}}\left(0.8\;/\;0.08\;-\;H\;/\;E\right)$$

$$dK\;/\;dt\;=\;k_{\mathrm{homeostasis}}\left(1\;-\;K\;/\;0.06\right)\;+\;k_{HK}\left(0.8\;-\;H\right)$$

$$dM\;/\;dt\;=\;k_{\mathrm{homeostasis}}\left(1\;-\;M\;/\;0.06\right)\;+\;k_{HM}\left(0.8\;-\;H\right)$$

$$\begin{array}{l} dE/dt\;=\;k_{\mathrm{homeostasis}}\left(1\;-\;E\;/\;0.08\right)\;+\;k_{\mathrm{HE}}\left(0.8\;-\;H\right)\;\\ \;+\;k_{\mathrm{ME}}\left(M\;/\;\left(H\;+\;K\;+\;M\;+\;E\right)\;-\;0.06\right) \end{array}$$

The simulation was performed using fourth-order Runge-Kutta integration with the time interval of 60 min over a period of 100 days, resulting in four-cell compartment curves. From these, simulated measurement curves were computed, i.e., volume (sum of all compartments), total macrophages (sum of macrophages and Kupffer cells), and Kupffer cells and angiogenesis (derivative of endothelial growth). The six parameters were iteratively adjusted until simulated and measured curves matched.

### Statistical Analysis

Statistical analysis was performed using Prism 5.0 (GraphPad software). Results are shown as mean ± standard deviation. All statistical analyses were performed using one-way analysis of variance (ANOVA) followed by Bonferroni correction for multiple comparisons (*p < 0.05, **p < 0.01, ***p < 0.001).

## Results

### *In vitro* Binding Specificity of the Near-Infrared Fluorescent CD68 Probe

**Figure 1:**
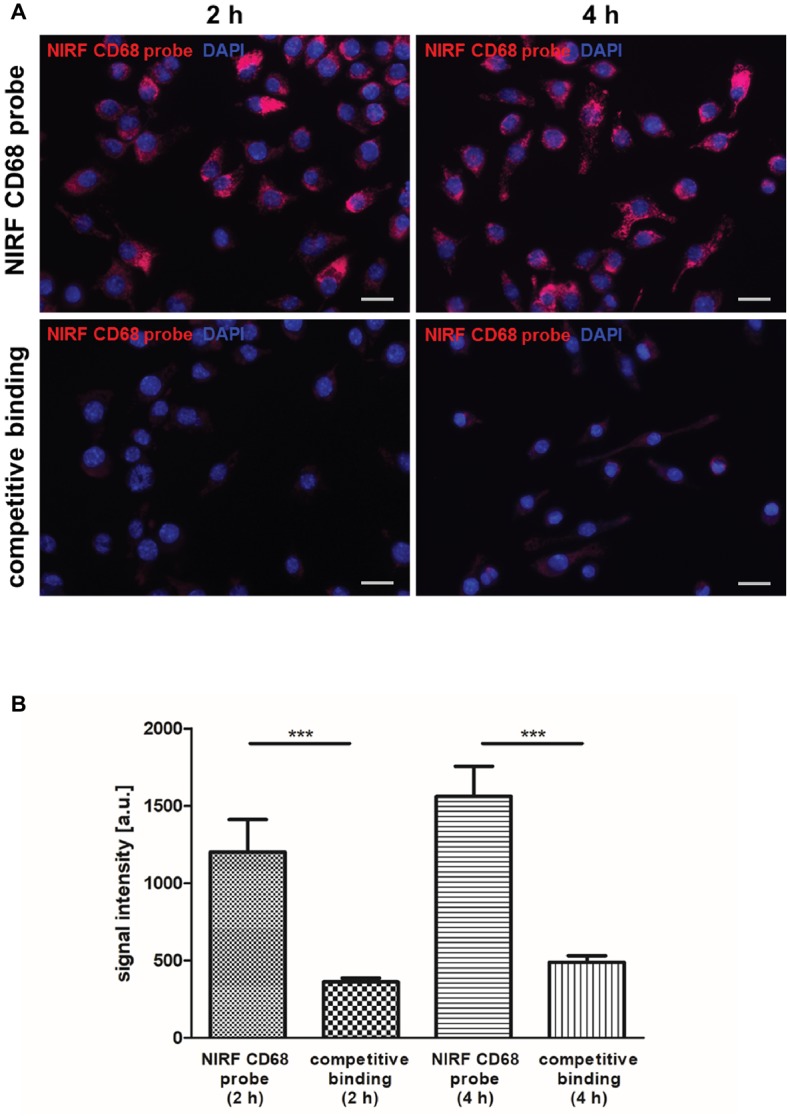
*In vitro* competitive binding analysis of the NIRF CD68 probe. To test the binding specificity of the NIRF CD68 probe, J774A.1 macrophages were incubated for 2 and 4 h with the NIRF CD68 probe alone (NIRF CD68 probe) or with the NIRF CD68 probe and a 10-fold molar excess of unlabeled anti-CD68 antibody (competitive binding). **(A)** Fluorescence images show a high fluorescent signal at 695 nm in macrophages after 2 and 4 h of incubation with the NIRF CD68 probe. Competitive binding results in a strongly reduced signal. NIRF CD68 probe in red, counterstaining of the nuclei with DAPI in blue, scale bar = 20 μm. **(B)** Quantitative analysis of mean fluorescent signal intensities in the NIRF CD68 probe group and in the competitive binding group shows a significantly lower signal intensity as a result of competitive binding (^***^p < 0.001; data are presented as mean values ± SD). a.u., arbitrary units.

For non-invasive imaging of macrophages by FMT-μCT, a NIRF probe targeting CD68^+^ macrophages was generated and evaluated *in vitro* and *in vivo*. The binding specificity of the NIRF CD68 probe was evaluated *in vitro* by competitive binding analysis using the macrophage cell line J774A.1. After 2 and 4 h of incubation with the NIRF CD68 probe alone, a strong fluorescent signal in the cells was observed (Figure 1A). Incubation of the cells with the NIRF CD68 probe together with a 10-fold excess of unlabeled anti-CD68 antibody resulted in a strongly reduced signal at both time points. Quantification of the fluorescent images revealed an increase in signal intensity from 2 to 4 h and confirmed a significantly lower mean signal intensity in the cells of the competitive binding group as compared to the cells incubated with the NIRF CD68 probe alone (*p* < 0.001) (Figure 1B).

### Biodistribution and *in vivo* Specificity of the Near-Infrared Fluorescent CD68 Probe

**Figure 2:**
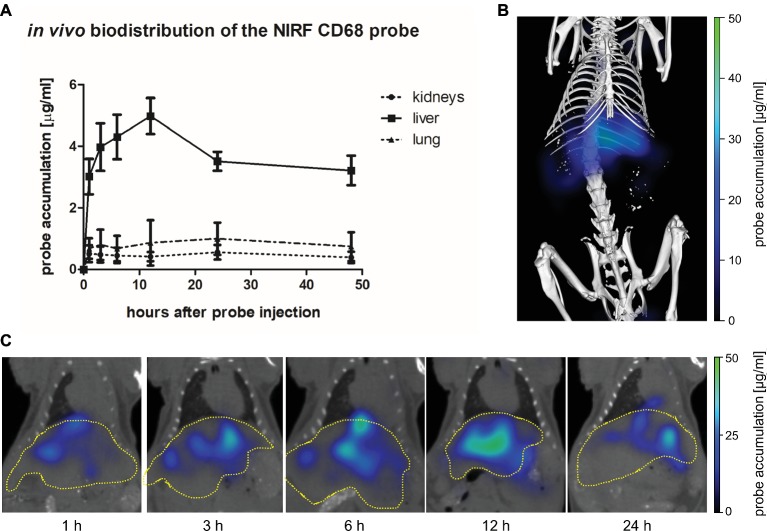
*In vivo* biodistribution of the NIRF CD68 probe. To analyze the biodistribution of the NIRF CD68 probe, the accumulation in different organs was measured longitudinally over 48 h in healthy mice by FMT-μCT. **(A)** Quantitative analysis of the NIRF CD68 probe concentration in the kidneys, liver, and lung revealed a significantly higher concentration in the liver compared to the kidneys and the lung at all measuring time points (*p* < 0.001 for each time point, data are presented as mean values ± SD). **(B)** Representative 3D rendering of reconstructed FMT-μCT data of a mouse 12 h after NIRF CD68 probe injection. **(C)** Representative frontal plane images of reconstructed FMT-μCT data at different time points after NIRF CD68 probe injection show an increasing fluorescent signal intensity in the liver until 12 h after injection, followed by a decline (dashed yellow line indicates the liver).

The biodistribution of the NIRF CD68 probe was analyzed longitudinally in healthy mice by FMT-μCT 1, 3, 6, 12, 24, and 48 h after injection. Quantitative analysis of the NIRF CD68 probe accumulation in the liver, lung, and kidneys revealed a significantly higher mean concentration in the liver as compared to the kidneys and the lung at all measuring time points (*p* < 0.001 for all time points, respectively) (Figure 2A). The concentration in the kidneys and the lung was constantly low without significant changes over time. In the liver, the mean concentration increased after probe injection reaching a maximum concentration at 12 h after injection. Thereafter, the mean probe concentration in the liver declined to a value similar to that observed at 1 h after injection (Figures 2A,C; representative 3D rendering of reconstructed FMT-μCT data shown in Figure 2B).

**Figure 3:**
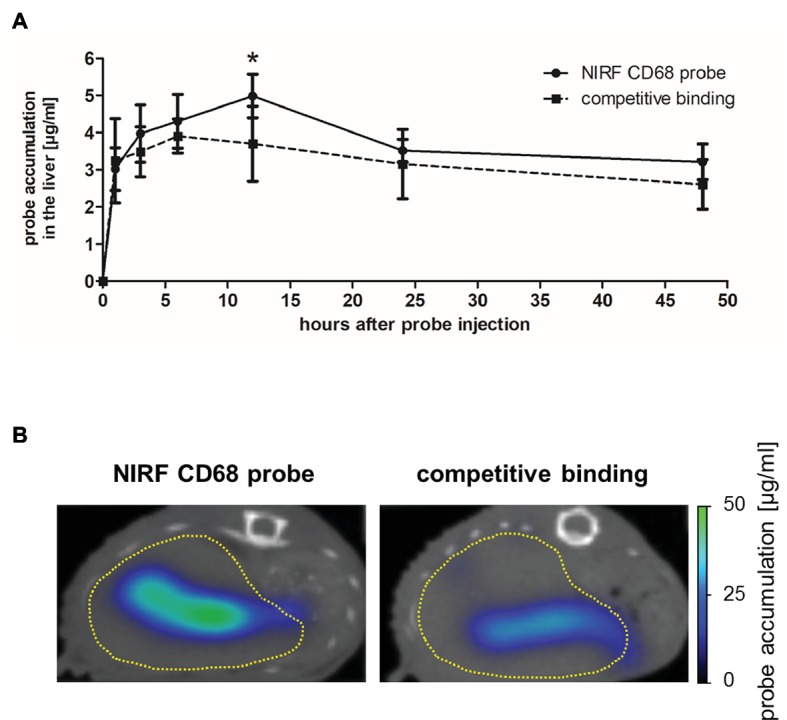
*In vivo* specificity of the NIRF CD68 probe. To analyze the *in vivo* specificity of the NIRF CD68 probe, a competitive binding experiment was performed in which a 5-fold excess of unlabeled CD68 antibody was injected 1 h prior to the injection of the NIRF CD68 probe (competitive binding group). Probe accumulation in the liver was compared to the concentration measured after injection of the NIRF CD68 probe alone (control group). **(A)** Quantitative analysis of probe accumulation in the liver showed a significant difference in the mean concentration 12 h after injection (^*^p < 0.05; data are presented as mean values ± SD). **(B)** Transversal FMT-μCT fusion images of representative mice 12 h after injection show a high fluorescent signal in the liver of the control mouse and a strongly reduced signal in the liver as a result of competitive binding (dashed yellow line indicates the liver).

The *in vivo* specificity of the NIRF CD68 probe was analyzed in a competitive binding experiment in which a 5-fold excess of unlabeled CD68 antibody was injected 1 h prior to injection of the NIRF CD68 probe (competitive binding group). In the liver, starting from 3 h after probe injection, a lower mean probe concentration was measured in the competitive binding group at each time point compared to the concentration measured after injection of the NIRF CD68 probe alone (control group) (Figure 3A; representative transversal FMT-μCT fusion images of competitive binding and control mice are shown in Figure 3B). The difference in the mean concentration of NIRF CD68 probe in the liver was significant 12 h after injection.

### Volumetric Recovery of the Liver After Partial Hepatectomy

**Figure 4:**
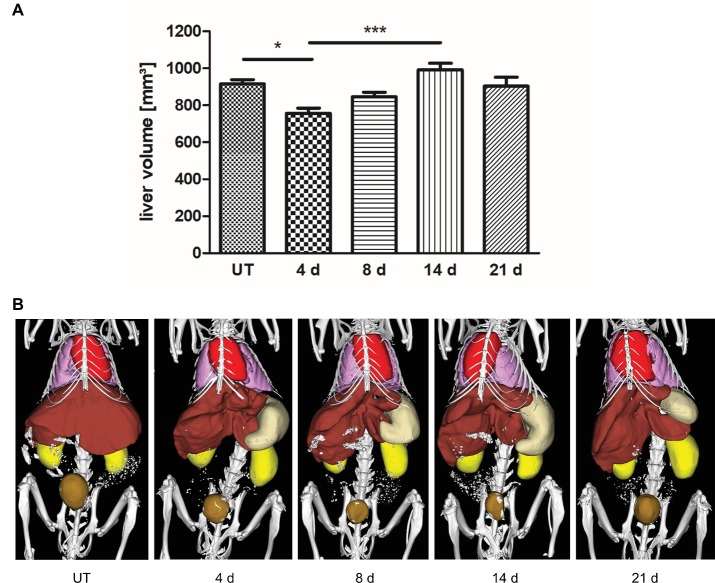
Liver volume recovery after PHx. Liver volume was determined non-invasively at day 4, 8, 14, and 21 after PHx and in untreated control mice by contrast-enhanced μCT. **(A)** Quantitative analysis showed that >70% and > 80% of the total liver volume were regained at day 4 and day 8 after PHx, respectively (^*^p < 0.05, ^***^p < 0.001; data are presented as mean values ± SD). **(B)** Representative 3D rendered CT images with segmented organs of an untreated control mouse and mice after PHx show the growth of the remaining liver lobes during regeneration (red, heart; pink, lungs; brown, liver; beige, stomach; ochre, bladder). UT, untreated mice.

To investigate liver regrowth after PHx by non-invasive imaging, the volume of the liver was measured *via* contrast-enhanced μCT. Quantitative analysis revealed that >70% of the total liver volume was reached at day 4 and > 80% was regained at day 8 after PHx (Figure 4A; representative 3D rendered CT images with segmented organs of an untreated control mouse and mice after PHx are shown in Figure 4B). The mean value determined at day 14 was slightly above the mean volume of the liver of untreated control mice. The mean volume at day 21 after PHx was comparable to control values.

### *In vivo* Monitoring of CD68^+^ Macrophage Density During Liver Regeneration

**Figure 5:**
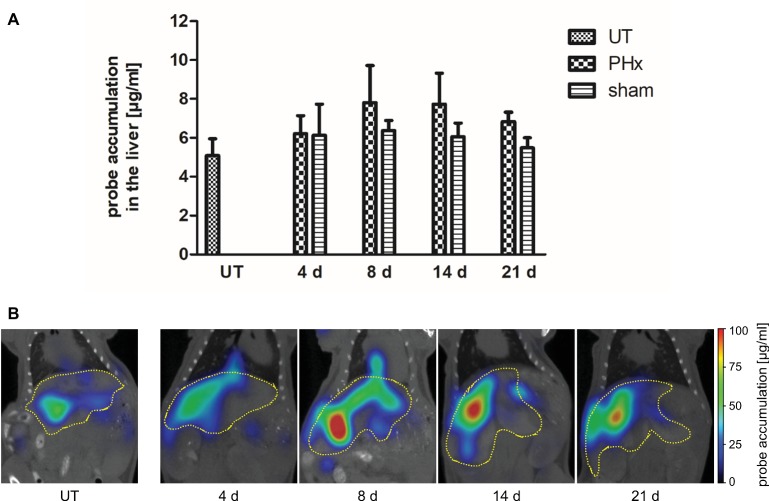
*In vivo* assessment of CD68^+^ macrophages in untreated control mice, sham-operated mice and mice after PHx. CD68^+^ macrophages were non-invasively monitored by FMT-μCT at day 4, 8, 14, and 21 after PHx and sham surgery and in untreated control mice. **(A)** Quantitative analysis showed a higher mean NIRF CD68 probe concentration in the liver at day 8, 14, and 21 after PHx compared to untreated and sham-operated control mice. The highest mean concentration was reached at day 8 (data are presented as mean values ± SD). **(B)** Representative frontal plane FMT-μCT fusion images of an untreated control mouse and mice after PHx show a higher fluorescent signal in the liver of mice at day 8, 14 and 21 after PHx (dashed yellow line indicates the liver). UT, untreated mice.

Macrophages play an important stimulatory role during liver regeneration (Takeishi et al., 1999; Abshagen et al., 2007; Nishiyama et al., 2015). Thus, we investigated the time course of macrophage density after PHx by non-invasive FMT-μCT imaging. CD68^+^ macrophages were monitored at day 4, 8, 14, and 21 after PHx and sham surgery using the NIRF CD68 probe. As an additional control, untreated mice were measured. Quantitative analysis of probe accumulation revealed a higher mean NIRF CD68 probe concentration in the liver at day 8, 14, and 21 after PHx compared to untreated and sham-operated control mice (Figure 5A). The mean concentration was highest at day 8 and 14 after PHx followed by a decline between day 14 and 21 indicating a transient increase in the density of CD68^+^ macrophages. No major changes in the mean NIRF CD68 probe concentration were measured in the liver of sham-operated mice during the whole observation period. Representative frontal plane FMT-μCT fusion images of an untreated control mouse and mice after PHx are shown in Figure 5B.

### Immunohistochemical Analysis of Macrophage Subpopulations During Liver Regeneration

**Figure 6:**
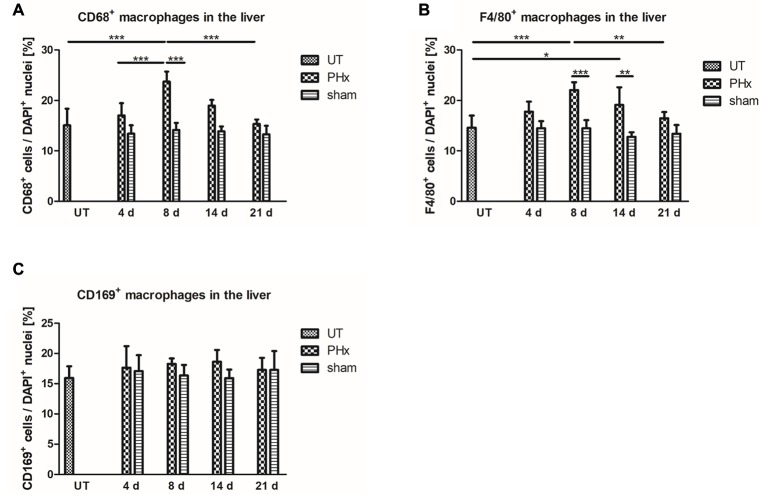
Immunohistochemical analysis of CD68^+^, F4/80^+^ and CD169^+^ macrophages in the liver of untreated and sham-operated control mice and mice after PHx. The density of CD68^+^, F4/80^+^, and CD169^+^ macrophages was determined by immunohistochemical analyses in liver sections taken from mice at day 4, 8, 14, and 21 after PHx and sham surgery and from untreated control mice (^*^p < 0.05, ^**^p < 0.01, ^***^p < 0.001; data are presented as mean values ± SD). **(A,B)** Quantitative analysis of the density of CD68^+^ **(A)** and F4/80^+^ **(B)** macrophages showed an increase in the mean values after PHx with a maximum at day 8. **(C)** Quantitative analysis of the density of CD169^+^ macrophages showed no significant differences in the liver of untreated and sham-operated control mice and mice after PHx. UT, untreated mice.

To validate the *in vivo* results, the density of CD68^+^ macrophages in the liver of untreated and sham-operated control mice and mice after PHx was determined by immunohistochemical analyses. Quantification of the density of CD68^+^ macrophages confirmed the trend of the *in vivo* findings showing a significant increase in the mean values after PHx until day 8 followed by a decline to levels observed in untreated control animals at day 21 (Figure 6A, *p* < 0.001). The mean density in the liver of sham-operated animals remained similar to that of untreated control animals without significant changes over time.

For further validation, we performed an immunohistochemical analysis of cells expressing F4/80, a generic macrophage marker that is independent of CD68 expression. Quantitative analysis of the density of F4/80^+^ macrophages showed a similar trend over time as compared to CD68^+^ macrophages with a significant transient increase and highest mean values at day 8 after PHx (Figure 6B, *p* < 0.001). Again, the mean density of F4/80^+^ in the liver of sham-operated animals was comparable to that of untreated control animals and did not significantly change over time.

An increased density of macrophages during liver regeneration can be a result of tissue-resident Kupffer cell proliferation or caused by an infiltration of macrophages from the blood circulation. Both Kupffer cells and infiltrating macrophages have been shown to play an important role during liver regeneration (Takeishi et al., 1999; Abshagen et al., 2007; Melgar-Lesmes and Edelman, 2015; Nishiyama et al., 2015). To investigate the contribution of tissue-resident macrophages to the increased macrophage density, we analyzed the expression of CD169 by immunohistochemical analysis. In contrast to CD68^+^ and F4/80^+^ macrophages, no significant differences in the density of CD169^+^ macrophages were observed in the liver of untreated and sham-operated control mice and mice after PHx (Figure 6C).

### Immunohistochemical Analysis of Angiogenesis During Liver Regeneration

Angiogenesis is a crucial process involved in liver regeneration, and macrophages have been shown to stimulate endothelial cell activation and to regulate vessel growth (Melgar-Lesmes and Edelman, 2015). To investigate the interrelation between macrophage density and angiogenesis, we analyzed the microvessel density and angiogenic activity in the liver of untreated control mice and in mice after PHx and sham surgery using immunohistochemistry.

**Figure 7:**
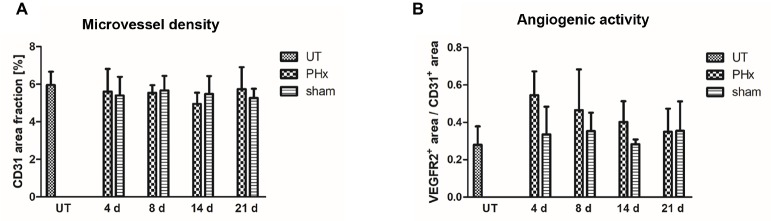
Immunohistochemical analysis of the microvessel density and angiogenic activity in the liver of untreated and sham-operated control mice and mice after PHx. The microvessel density and angiogenic activity were determined by immunohistochemical analysis of liver sections taken from mice at day 4, 8, 14, and 21 after PHx and sham surgery and from untreated control mice (data are presented as mean values ± SD). **(A)** Quantitative analysis of the microvessel density showed no significant differences between untreated and sham-operated control mice and mice after PHx. **(B)** Quantitative analysis of the angiogenic activity showed a markedly increased mean activity at day 4 after PHx that decreased steadily until day 21. UT, untreated mice.

Microvessel density was not significantly different between mice after PHx, untreated and sham-operated control mice (Figure 7A). However, the angiogenic activity, as assessed by the proportion of the VEGFR2^+^ area fraction to the CD31^+^ area fraction, was markedly increased on day 4 after PHx followed by a continuous decrease until day 21 (Figure 7B). In sham-operated and untreated mice, no significant changes were found over time.

### Modeling of Liver Regeneration After Partial Hepatectomy

**Figure 8:**
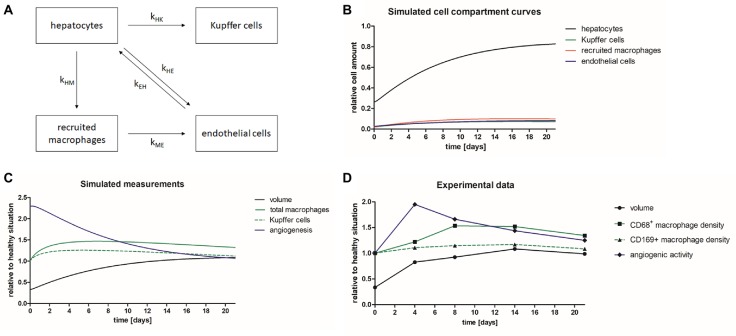
Mathematical model describing liver regeneration. A mathematical model has been developed describing liver regeneration at organ and tissue scale. **(A)** The scheme shows the interrelations between liver cell compartments (hepatocytes, Kupffer cells, recruited macrophages, and endothelial cells) during liver regeneration. **(B)** The simulated cell compartment curves show the normalization of relative cell amounts after PHx. **(C)** The simulated measurement curves were computed based on the simulated cell compartment curves. After iterative adjustment the simulated curves describing liver volume, total macrophages, Kupffer cells, and angiogenesis showed similar time courses as the experimental data. **(D)** Diagram showing the time courses for liver volume, CD68^+^ macrophage density, CD169^+^ macrophages, and angiogenic activity of the experimental data acquired in mice after PHx.

The *in vivo* and immunohistochemical results revealed different time courses of volume recovery, macrophage density and endothelial cell activation (angiogenesis). A simplified mathematical model was developed including different cell compartments of the liver (hepatocytes, Kupffer cells, recruited macrophages, and endothelial cells), and the growth and interplay of these compartments after PHx was simulated (Figures 8A,B). Based on the resulting cell compartment curves (Figure 8B), simulated measurement curves were computed describing liver volume (sum of all compartments), total macrophages (sum of recruited macrophages and Kupffer cells), and Kupffer cells and angiogenesis (derivative of endothelial growth) (Figure 8C). After parameter adjustment, the time courses of the numerical model matched the experimental data of angiogenesis, macrophages, and liver volume obtained by non-invasive imaging and immunohistochemical analyses (Figures 8C,D). In detail, there was an early onset of angiogenesis, which was followed by an increase in the overall macrophage density peaking on day 8. The liver volume increased rapidly after PHx reaching levels above healthy liver on day 14 before normalization.

## Discussion

Liver regeneration after injury or partial resection comprises a complex interplay of different cell types and is tightly regulated at various scales (Taub, 2004; Michalopoulos, 2010; Li and Hua, 2017). To address alterations during liver regeneration after PHx at different levels, we used non-invasive imaging in combination with immunohistochemistry and developed a simple mathematical model describing the interrelations between different cell types involved in liver regeneration, angiogenesis and liver volume recovery.

Liver volume recovery was non-invasively monitored by μCT imaging. The measurements revealed that >70% of the total liver volume were regained within 4 days. However, normalization of the liver volume was not reached until day 21 due to an increased volume observed on day 14. The increased volume can be explained by edema formation that sometimes occurs during liver regeneration (Pleskovic et al., 1996).

Since macrophages are known to play an important stimulatory role during liver regeneration, we investigated the density of different macrophage populations after PHx by non-invasive imaging and immunohistochemistry. For non-invasive imaging, we used combined FMT-μCT imaging and generated a NIRF probe targeting CD68. Specific binding of the NIRF probe to CD68^+^ macrophages was confirmed by competition analyses *in vitro* and *in vivo*. Quantitative *in vivo* FMT-μCT imaging after PHx and sham surgery revealed an increased mean concentration of the CD68 probe in the liver at day 8 and 14 after PHx indicating a transient increase in the density of CD68^+^ macrophages. Immunohistochemical analyses of CD68^+^ and F4/80^+^ macrophages showed the same trend with a significantly higher macrophage density on day 8 after PHx as compared to untreated and sham-operated control mice. However, the immunohistochemical data were more distinct than the results obtained by FMT-μCT imaging. The difference in precision can be explained by the lower spatial resolution of the FMT (about 2 mm). Small changes in the density of macrophages are more difficult to determine by FMT-μCT than *via* immunofluorescence microscopy. Furthermore, although absorption and scattering of the photons are taken into account in the fluorescence reconstruction algorithms, numerical limitations of the complex diffuse optical behavior can still affect the accuracy of the data, especially in deeper lying organs and in organs with a high blood volume, such as the liver, since blood is the main near-infrared absorber *in vivo* (Gremse et al., 2014). Moreover, the blood pool of the circulating probe and the unspecific hepatic uptake of foreign substances such as probes and contrast agents can lead to an unspecific probe signal. Antibodies are known to have a long blood half-life (Freise and Wu, 2015). Since we used an antibody as targeting molecule, we cannot exclude that the blood pool of the imaging probe has influenced the measurements. To reduce the impact of the blood pool, a different targeting molecule could be chosen, e.g., a nanobody, that has a shorter blood half-life.

To analyze the contribution of tissue-resident Kupffer cells in liver regeneration after PHx, we investigated the expression of CD169 by immunohistochemical analysis. Quantification revealed no significant differences in the density of CD169^+^ macrophages in the liver of untreated and sham-operated control mice and mice after PHx. Thus, while the overall density of macrophages in the liver increased significantly after PHx, the density of tissue-resident CD169^+^ Kupffer cells did not. This is in line with previous findings showing that the number of Kupffer cells correlates with liver restoration rate (Melgar-Lesmes and Edelman, 2015). Therefore, the results provide further evidence for the involvement of macrophages recruited from the blood circulation in liver regeneration after PHx (Melgar-Lesmes and Edelman, 2015; Nishiyama et al., 2015; Wen et al., 2015). However, further investigation is needed to unravel the details of resident Kupffer cell and infiltrating macrophage contribution to liver regeneration.

Angiogenesis is an important process involved in liver regeneration after hepatectomy and mutual interactions have been described between hepatocytes, Kupffer cells/macrophages and endothelial cells during liver regeneration (Drixler et al., 2002; Uda et al., 2013; Castiglione et al., 2014). Therefore, we analyzed the angiogenic endothelial cell activity and microvessel density in the liver after PHx by immunohistochemistry. A markedly increased mean angiogenic activity was detected at day 4 after PHx that decreased steadily until day 21 which is in accordance with previously published data (Alizai et al., 2017). Quantitative analysis of the microvessel density showed no significant differences between mice after PHx and control mice. This finding is not in line with results published by other groups that showed an increase in the microvessel density following PHx (Drixler et al., 2002, 2003). The discrepancy can be explained by different quantification methods. While we included larger arterioles and venules in the quantification, they were excluded by the other groups. Larger vessels contribute more to the overall CD31^+^ area fraction than very small vessels. Thus, changes in the density of these small vessels do not have a major effect on the overall vessel density.

The *in vivo* and immunohistochemical results revealed differences in the time courses for volume recovery, macrophage density and angiogenic endothelial cell activity, nevertheless, all three time courses show a progression towards levels of healthy situation over time. To describe the interrelation between volume recovery, macrophage density and angiogenesis occurring at different scales, we developed a numerical model that describes the growth and interplay of the involved liver cell compartments (hepatocytes, Kupffer cells, recruited macrophages, and endothelial cells). A numerical model serves to bridge the gap between hidden parameters (e.g., *k*_HE_) and observable measurements. The model may contain many direct interactions between cell types which are simple by themselves but result in a complicated situation altogether, which cannot be described by closed formulas but require numerical approaches instead. Our simulated measurements generally reflect the experimental data obtained by non-invasive imaging and immunohistochemical analyses. Differences remain in the earlier increase of the macrophage and Kupffer cell populations in our model as compared to the measurements. The maximum in angiogenic activity precedes the peak of macrophage density and normalization of liver volume. This shows that the model can describe liver regeneration at organ and tissue scale, and that the model substantially benefits from experimental quantitative non-invasive imaging data. Nevertheless, higher sample numbers would improve the stability and reliability of the model. At tissue scale, different mathematical models with considerable higher complexity than our model have been established that describe and predict specific important processes involved in liver regeneration. Recently, a mathematical model revealed a crucial role of hybrids consisting of hepatocytes and bone marrow cells that trigger proliferation in the regeneration process (Pedone et al., 2017). For liver regeneration after CCl_4_ damage, Hoehme et al. have established a model that describes structural alignments of hepatocytes to sinusoids as a crucial pre-requisite for regaining the complex microarchitecture (Hoehme et al., 2010). Simple algorithmic models like the one proposed here have the advantage of high robustness and thus the suitability for integrating less quantitative *in vivo* data but also face several limitations. The model does not comprise the full complexity of the interrelations between the hepatocytes, macrophages, and endothelial cells that occur during liver regeneration. In addition, it does not describe causal relationships between the involved cell compartments in a detailed mechanistic way. Furthermore, the model does not take into consideration hepatic stellate cells, resident and monocyte-derived liver macrophages, additional immune cells such as lymphocytes or dendritic cells, the biliary system, different blood vessel compartments, hepatic blood flow or portal vein pressure, or the complex microarchitecture of the liver. Nevertheless, our model links information about liver regeneration and the interaction of different cell compartments (hepatocytes, Kupffer cells/macrophages, endothelial cells) from tissue to organ scale data. While our model is simplification as explained above, it can be extended to describe further liver cell compartments such as stellate cells, bile duct cells, and additional immune cells beyond macrophages or different macrophage populations. In addition, hepatocyte subpopulations in different activation states such as quiescent, primed, and replicating cells could be included as described by Furchtgott et al. (2009). A numerical model can also be used to extrapolate additional time points. To enable unambiguous parameter estimation, an increase in model complexity should be accompanied by an increase of measurement values. In our study, we used *in vivo* and immunohistochemical analyses for modeling, resulting in mean time curves and therefore one model per group. If longitudinal *in vivo* measurements are used, a model could be applied to analyze individual mice, enabling statistical comparison of kinetic parameters between groups. Either way a numerical model could be used to investigate and explain the effects of genetic modifications, e.g., *csf1-*knock-out resulting in a reduced number of macrophages (Amemiya et al., 2011), macrophage depletion, or extended liver resection (Christ et al., 2017) on different aspects of liver regeneration and such data could be used to refine, extend, or validate our simplified model. In addition, advanced numerical models could be used to get a comprehensive insight into the interrelations between different cells and signaling pathways in chronic liver disease progression or in response to therapeutic interventions (Cook et al., 2015; Schwen et al., 2016).

In summary, based on non-invasive imaging and immunohistochemical analyses, we have established a mathematical model for liver regeneration describing the interrelations between hepatocytes (volume recovery), macrophages, and endothelial cells (angiogenesis) at organ and tissue scale. In this context, non-invasive imaging and suitable probes targeting cell populations such as macrophages are of great value for data acquisition in the course of liver regeneration at organ scale.

## Ethics Statement

All animal experiments were performed according to German legal requirements and animal protection laws and were approved by the Authority for Environment Conservation and Consumer Protection of the State of North Rhine-Westphalia (LANUV).

## Author Contributions

SZ and AM performed the experiments, acquired, and analyzed the data and wrote the manuscript. AR and DD participated in the experiments and data analyses. FG performed the modeling and FMT-μCT reconstruction. SW participated in the *in vivo* experiments. JFB participated in probe generation. UA participated in supervision and study design. JGB participated in study design. FK participated in study design, provided supervisory support, and edited the manuscript. WL designed and coordinated the experiments, provided supervisory support, wrote, and edited the manuscript. All authors read and approved the final version of the paper. SZ and AM contributed equally to this work.

### Conflict of Interest Statement

The authors declare that the research was conducted in the absence of any commercial or financial relationships that could be construed as a potential conflict of interest.
